# Using a Technology Acceptance Model to Explore the Intention to Use Digital Health Technologies Among People With Disabilities: Cross-Sectional Survey Study

**DOI:** 10.2196/79595

**Published:** 2025-11-20

**Authors:** Jae-Hak Kim, Janghyeon Kim, Bo-Young Youn

**Affiliations:** 1 Department of Fitness Promotion and Rehabilitation Exercise, National Rehabilitation Center, Seoul, Republic of Korea; 2 Department of Style-Tech, Hwasung Medi-Science University, Hwaseong-si, Gyeonggi-do, Republic of Korea; 3 Department of Healthcare Management, College of Health and Medical Science, Daejeon University, #505, Moonmugwan, 62, Daehak-ro, Dong-gu, Daejoen, 34520, Republic of Korea, 82 42-280-4096

**Keywords:** technology acceptance model, digital health, disability, health management, intention to use, digital health literacy, people with disabilities

## Abstract

**Background:**

Electronic personal health records (e-PHRs) can improve health management; however, people with disabilities face adoption barriers. Identifying acceptance drivers in this population is essential.

**Objective:**

This study aims to determine factors shaping intention to use e-PHRs among people with disabilities within a technology acceptance model (TAM) framework, including external determinants (health consciousness [HC], health information consent [HIC], content characteristics [CC], information security [IS], eHealth literacy [eHL], and effectiveness [EF]).

**Methods:**

A nationwide survey of people with disabilities in South Korea (N=800) was conducted across rehabilitation hospitals, disability welfare centers, and public health centers (August 30 to November 30, 2023) using proportionate stratified and systematic stratified cluster sampling. Hypotheses were tested via structural equation modeling with bootstrapped mediation (2000 resamples) and multigroup analyses by disability severity.

**Results:**

Usage intention (UI) was primarily driven by perceived usefulness (PU; β=0.662; *P*<.001) and additionally by perceived ease of use (PEU; β=0.203; *P*<.001). Ease of use increased usefulness (β=0.452; *P*<.001). External predictors of PEU were HC (β=0.233; *P*<.001), CC (β=0.163; *P*<.001), HIC (β=0.167; *P*<.001), IS (β=0.089; *P*=.005), and EF (β=0.276; *P*<.001); eHL was not significant (β=0.025; *P*=.41). Predictors of PU were EF (β=0.368; *P*<.001) and HIC (β=0.243; *P*<.001), while CC (β= −0.121; *P*=.002) and eHL (β= −.068; *P*=.003) were negative; HC and IS were not significant. Indirect effects supported PEU→PU→UI (β_indirect=0.299; 95% CI 0.210‐0.404). The largest total upstream effects on associations with intention were EF (β_total=0.382; *P*<.001) and HIC (β_total=0.245; *P*<.001). Multigroup structural equation modeling (mild, n=432; severe, n=368) indicated PU was a stronger driver of intention in the mild group (β=0.727) than the severe group (*β*=.511). PEU also contributed (severe β=0.272; mild β=0.171). CC predicted PEU only in the mild group (β=0.201; *P*<.001), whereas IS predicted PEU only in the severe group (β=0.119; *P*=.003).

**Conclusions:**

This study highlights that PU and PEU are crucial mediators driving the adoption of e-PHR among people with disabilities. These findings suggest the need for designing user-friendly digital health solutions that integrate robust support systems, address privacy concerns, and deliver high-quality, relevant content tailored to this population. The restriction to people with disabilities using rehabilitation, public health, or welfare centers introduces selection bias. Future studies should broaden sampling to include a diverse population.

## Introduction

### Background

The digital transformation of health care industries worldwide has been dedicated to promoting initiatives that aim to improve human health and quality of life [1]. In South Korea, digital health care is increasingly integrated into various health care services, encompassing personal health records, mobile health, health information technology, wearable devices, telehealth and telemedicine, personalized medicine, and digital therapeutics. These comprehensive approaches empower consumers by enabling them to independently manage and control health and well-being [2]. Particularly, electronic personal health records (e-PHRs) refer to systems where individuals centrally manage and integrate lifelong health information and selectively share it with chosen recipients [3]. Such systems have gained attention for the utmost potential to provide valuable data for personalized health care services, thereby addressing societal health challenges [4]. Furthermore, the usage of personal health records within the health care sector has expanded into various domains, including personal medical device–linked health management services and public health care systems. With advancing technology, this usage is increasingly extended to wearable devices [5].

Globally, e-PHRs have become increasingly important as the Fourth Industrial Revolution accelerated the expansion of remote medical services, prompting the development of consumer-centered, effective health management solutions [6]. In particular, global IT corporations are playing pivotal roles in establishing and advancing the digital health care ecosystem through devices and platform technologies. Prominent international examples include the “Blue Button” service introduced by the United States Department of Veterans Affairs in 2010, Australia’s “My Health Record” managed by the Australian Digital Health Agency, and Canada’s establishment of regional health integration networks to facilitate coordination among health care providers and care centers. Additionally, countries such as Sweden, Norway, the Netherlands, France, Germany, Australia, and Singapore have undertaken significant efforts to develop platforms that enable individuals to access and use personal health data easily [7].

However, as remote interactions become commonplace and living environments rapidly transition to an online-centered context, people with disabilities face significant challenges in adapting to these changes due to limitations in daily activities and the accelerating pace of digital transformation [8]. If smart health care environments are designed without taking into account the different types of disabilities and physical limitations, people with disabilities may face significant challenges in using these devices effectively [9,10]. Furthermore, when offering telemedicine services to individuals with disabilities, issues related to language, cognitive abilities, and sensory limitations can hinder effective communication between users and health care providers, thus highlighting and worsening inequalities in digital accessibility [11]. Therefore, addressing health rights issues among people with disabilities necessitates heightened social attention and proactive integration of digital health care services. Furthermore, comprehensive health information management, starting from hospital-based care and extending through rehabilitation services, must be linked systematically with community-based support networks to effectively meet the health care needs of people with disabilities [12].

Recent studies indicate that numerous developed welfare states, including those in Europe, the United States, and Japan, are actively implementing e-PHRs to promote healthier individuals and societies [13,14]. With the paradigm shift from hospital- and health care provider-centered care to patient-centered approaches, driven by the integration of medical information and scientific technologies, comprehensive data, including lifestyle habits, medical information, and patient emergency conditions, are increasingly used for both treatment and preventive health care [15]. Currently, e-PHR services are primarily implemented in standalone formats through health care institutions, using mobile apps that provide individuals with basic personal health information, including appointment scheduling, health screenings, medication details, and laboratory results [16].

However, unlike people without disabilities, those with disabilities frequently interact with multiple institutions, such as hospitals, public health centers, disability welfare centers, and rehabilitation facilities, from the onset of disability onward for treatment, rehabilitation, and health management [17]. Given that information provided solely through medical institutions is limited for comprehensive health management, there is an urgent need to emphasize community-based rehabilitation approaches and develop integrated e-PHR services to facilitate holistic health care management for people with disabilities [18].

To address these challenges, it is necessary to investigate the causal relationships among external variables influencing the intention to use e-PHRs for health management among people with disabilities, using the technology acceptance model (TAM) framework [19]. Additionally, comparative analyses of multiple structural models can further provide empirical insights into the practical implementation of e-PHR services for people with disabilities [20]. Understanding perceptions toward e-PHR services among people with disabilities is fundamental not only to improving the quality of life but also to enhancing broader social metrics, such as preventing secondary disabilities, managing chronic illnesses, and reducing medical costs, thereby contributing to the establishment of a healthier society. Emphasizing social values and fostering sustained resource development is crucial for collectively addressing social, psychological, and physical challenges encountered by people with disabilities. Therefore, this study aims to conduct a comprehensive survey among people with disabilities in South Korea, focusing on the usage of e-PHR services among various digital health care platforms to manage health effectively. Using structural equation modeling (SEM) based on the TAM, this study aims to identify and analyze service-related factors influencing the intention to use e-PHRs among people with disabilities, thereby predicting perceptions toward e-PHR services prior to the actual implementation and providing foundational data for future service development.

Although the TAM has been widely used in health care adoption research, previous studies focusing on people with disabilities typically have several shortcomings. These studies often (1) concentrate on single disability groups or small clinical cohorts [21], (2) overlook considerations of consent, security, and content quality that are essential for people with disabilities navigating fragmented care [22], and (3) treat digital skills as universally enabling rather than recognizing them as potentially critical or obstructive [23]. To address these gaps, this study expands on the foundational TAM framework (perceived ease of use [PEU] → perceived usefulness [PU] → usage intention [UI]) by incorporating 6 external factors that are relevant for people with disabilities. These factors include health information consent (HIC; willingness to share data amidst privacy concerns), information security (IS; trust in protective measures), content characteristics (CC; structure, clarity, and cognitive load), effectiveness (EF; perceived assistance and facilitating conditions across different institutions), health consciousness (HC), and eHealth literacy (eHL). Using a large, nationally representative sample of people with disabilities from various health institutions, this study tests several pathways and reveals some unexpected effects. This study expands the core TAM by incorporating 6 important external factors related to disability. By testing this model in a large, nationally representative sample, the research clarifies how design features, consent and security considerations, and supportive conditions influence acceptance among individuals with disabilities. Therefore, this study lays the foundation for developing more responsive e-PHR services that cater to the needs of people with disabilities.

### Research Model

**Figure 1.**
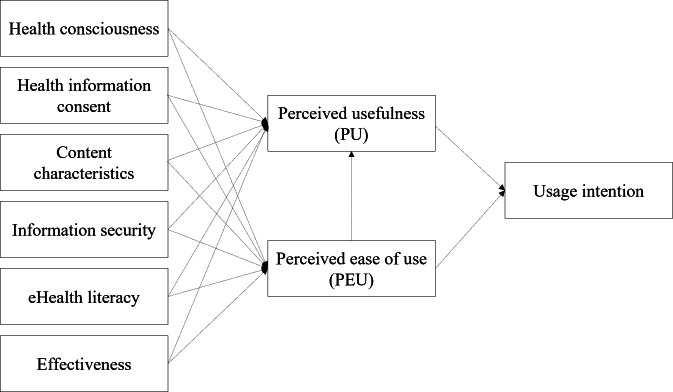
Conceptual research model using technology acceptance model constructs to assess digital health technology adoption for people with disabilities.

This study develops a research model based on the TAM, introduced by Davis [24], and integrates findings from prior research related to the intention to use digital health care services. The model incorporates 6 external factors, such as HC, consent to use health information, CC, IS, eHL, and EF. The primary objective is to investigate how these external factors influence UI through the mediating roles of PEU and PU. The proposed research model is illustrated in Figure 1.

## Methods

### Participants and Data Collection

This study conducted a nationwide survey as a foundational investigation into e-PHRs within the digital health landscape. The sampling methods used were proportionate stratified sampling and systematic stratified cluster sampling. The selection criteria included individuals aged 19 years or older with physical disabilities diagnosed at least 3 years prior, who had used or were currently using community-based institutions, such as public health centers or disability welfare centers. Participants were excluded from the study for the following reasons: (1) those with Mini-Mental State Examination (MMSE) scores below 24 were excluded to ensure cognitive ability sufficient to comprehend [25] and voluntarily participate in the survey, thereby enhancing the validity and reliability of the collected data; (2) individuals who were hospitalized for acute care, undergoing active surgery or treatment, or unable to complete the survey, for example, due to incomplete responses or withdrawal during participation, were also excluded to reduce bias related to acute clinical status and to ensure representativeness of stable, community-dwelling individuals with disabilities; and (3) surveys with missing data on key variables necessary for analysis were excluded according to established guidelines for missing data based on the “Statistical Analysis” section to ensure data integrity. These criteria were adopted to ensure that responses accurately reflected the experiences, intentions, and health conditions of the target population capable of meaningful engagement with digital health technologies for health management.

Recruitment took place across 3 community-based settings in South Korea, namely rehabilitation hospitals, disability welfare centers, and public health centers, from August 30 to November 30, 2023. Each participating site designated a gatekeeper who undertook the following tasks: (1) posted Institutional Review Board (IRB)–approved flyers in public areas and program rooms; (2) distributed large-print and plain-language information sheets during group sessions; and (3) provided assisted, standardized screening. To implement proportionate stratified and systematic cluster sampling, target quotas were allocated to strata defined by region (metropolitan vs medium or small cities) and type of institution. Within each stratum, sites (clusters) were selected, and on preselected recruitment days, staff approached every k-th eligible visitor (with k determined by expected daily traffic, typically ranging from 3 to 5) to minimize selection bias. A total of 1217 participants completed the survey; however, only 800 participants’ survey data (a response rate of 65.7%) were available for analysis.

### Sample Size Calculation

To determine an appropriate sample size, guidelines provided in prior literature were followed. According to Hair et al [26] and Kline [27], SEM requires at least 10-20 respondents per observed variable (questionnaire item) to ensure reliable parameter estimates and model fit. In this research, the measurement instruments comprised 73 observed variables across 9 latent variables. Based on these criteria, the required sample size was calculated as follows: the required minimum sample size was calculated by multiplying the number of observed variables (73) by the recommended minimum number of respondents per variable (10), resulting in a total of 730 respondents. Considering the need to secure sufficient statistical power and to address possible missing or invalid responses, the final sample size was set at 800 respondents. This figure comfortably exceeds the recommended sample size based on the number of observed variables, ensuring suitability for SEM analysis using AMOS (Analysis of Moment Structures).

### Measurement Instruments

The survey instrument was developed by modifying and refining existing measurement tools from previous literature and theoretical frameworks related to technology acceptance, ensuring alignment with the study’s objectives and context.

Since the sample included people with disabilities, all instruments were adapted in advance according to universal design principles to ensure accessibility while preserving the intended meaning. Accommodation was made for various needs, including (1) visual accommodation, such as large print materials and read-aloud options; (2) hearing accommodation, such as sign language interpretation or captioning; (3) motor accommodation that involved assistance with pointing or dictation and extended time for completion; and (4) cognitive or communication support through plain-language summaries and brief examples. Researchers also supported augmentative and alternative communication methods, such as tablet typing or pointing, and ensured that surveys could be completed privately, with adequate time provided for all participants. Administration followed a standardized protocol for completing assessments through self-administration or with the assistance of an interviewer, with assistants receiving brief training in disability etiquette, neutrality, and confidentiality. For each case, the mode of administration, type of assistance provided, and time taken to complete the assessment were recorded. Content validity was ensured through a pilot study focused on individuals with disabilities, during which a multidisciplinary panel—including a rehabilitation physician, a public health expert, and representatives from the community of persons with disabilities—reviewed the clarity and relevance of the assessment items, making necessary revisions. Cognitive debriefing, along with a small pilot study involving 10 individuals with physical disabilities, confirmed the assessment’s feasibility and understanding, resulting in minor changes to wording and response options. Following the above content validity testing and a pilot survey, the final questionnaire was established.

The TAM was used to explore intentions regarding the use of e-PHRs for health management among people with disabilities. The finalized survey comprised a total of 73 items organized into 9 categories, namely HC; 8 items, HIC (8 items), CC (6 items), IS (4 items), eHL (14 items), EF (16 items), PU (7 items), PEU (5 items), and intention to use (5 items). Detailed descriptions of the measurement instruments are presented in Multimedia Appendix 1.

HC generally refers to the level of active engagement and efforts directed toward health promotion and disease prevention, particularly using e-PHRs [28]. The measurement instrument was developed by modifying and refining items from the HC questionnaire originally used by Belloc and Breslow [29]. Responses were measured on a 5-point Likert scale ranging from 1 (strongly disagree) to 5 (strongly agree), with higher scores indicating greater engagement in preventive health behaviors, such as exercise and dietary management. Previous research reported an internal consistency reliability (Cronbach α) of 0.852. In this study, Cronbach α was 0.843, confirming reliability. Exploratory factor analysis (EFA) yielded a KMO (Kaiser-Meyer-Olkin) value of 0.787, indicating adequate sampling adequacy.

Given the exponential increase in personal health data in the digital age, HIC, specifically consent for data sharing, raises significant privacy concerns, necessitating broad social consensus [30]. The measurement instrument for HIC was developed by adapting and refining items based on the health and psychological theories used by Bowman et al [31], specifically addressing consent related to the disclosure of personal health information. The questionnaire used a 5-point Likert scale ranging from 1 (strongly disagree) to 5 (strongly agree). Higher scores indicated a greater willingness to disclose personal health information, such as health status, medical examinations, treatment details, and exercise information, to relevant stakeholders. The internal consistency reliability (Cronbach α) was 0.886 in previous research, while in this study, Cronbach α was 0.945, demonstrating strong reliability. EFA yielded a KMO value of 0.892, indicating excellent sampling adequacy.

CC in the context of digital technologies refers to the ability of users to freely access and use information through various platforms, including computers, mobile devices, and the internet [32]. The measurement instrument for CC was developed by modifying and refining items based on previous studies [33,34]. Responses were recorded using a 5-point Likert scale ranging from 1 (not helpful at all) to 5 (extremely helpful). Higher scores indicated greater perceived benefits derived from content attributes, such as mobility and personalization. Internal consistency reliability (Cronbach α) from previous research was 0.938, while in this study, Cronbach α was 0.949, demonstrating excellent reliability. EFA yielded a KMO value of 0.921, indicating strong sampling adequacy.

IS refers to the technical measures implemented to prevent corruption, alteration, or unauthorized disclosure of information. Issues related to personal data protection and security represent significant social concerns and act as barriers to the broader adoption of ITs [35]. In this study, IS pertains specifically to trust and beliefs regarding the protection and security of personal data within e-PHR services. The measurement instrument for IS was developed by modifying and refining items derived from prior research by van Houwelingen et al [36], Shareef et al [37], and Lee and Ham [38]. Responses were recorded on a 5-point Likert scale ranging from 1 (strongly disagree) to 5 (strongly agree). Higher scores indicated greater trust and confidence in IS, reflecting perceptions of higher safety, confidentiality, and reliability. The internal consistency reliability (Cronbach α) was 0.856 in previous research, and Cronbach α in this study was 0.931, indicating excellent reliability. EFA yielded a KMO value of 0.850, signifying strong sampling adequacy.

eHL refers to a comprehensive set of skills required for acquiring and effectively using basic health information within health care contexts. Additionally, it is recognized as a critical determinant of health outcomes, shaping health-related decisions and enabling predictive approaches to everyday health management [39]. The measurement instrument for eHL was developed by modifying and refining items from the eHL scale originally proposed by Nutbeam [40] and subsequently adapted for the Korean context by Chung et al. [41]. Responses were recorded on a 5-point Likert scale ranging from 1 (strongly disagree) to 5 (strongly agree), with higher scores indicating greater proficiency in eHL, a stronger understanding of health concepts, and a higher level of preventive health behaviors. Internal consistency reliability (Cronbach α) was 0.961 in previous research, and Cronbach α obtained in this study was 0.842, indicating satisfactory reliability. EFA yielded a KMO value of 0.894, signifying excellent sampling adequacy.

The EF provided by e-PHRs to people with disabilities measures the perceived helpfulness of applying this technology to disability-related health management [42]. In this study, an assistance degree specifically refers to perceptions regarding the utility and beneficial impact of e-PHR services on personal health management. The measurement instrument was developed by modifying and refining items based on the PHR System Functional Model R1 report by the Healthcare Information and Management System Society (HIMSS [43]). Responses were recorded using a 5-point Likert scale ranging from 1 (not helpful at all) to 5 (extremely helpful). Higher scores indicated a greater perceived level of utility and assistance from e-PHR services. Internal consistency reliability (Cronbach α) reported in previous research was 0.931, while the reliability for this study was confirmed with a Cronbach α of 0.972, indicating excellent reliability. EFA yielded a KMO value of 0.943, demonstrating robust sampling adequacy.

PU refers to the degree to which individuals believe that using health management services can effectively enhance health outcomes and improve management efficiency [44]. The measurement instrument was developed by modifying and refining items based on the original constructs proposed by Venkatesh and Davis [45] and further adapted by Choi et al. [33]. Responses were recorded using a 5-point Likert scale ranging from 1 (strongly disagree) to 5 (strongly agree). Higher scores indicated stronger perceptions that e-PHR services would offer efficient and beneficial assistance. Previous research reported internal consistency reliability (Cronbach α) of 0.899, and this study confirmed excellent reliability with a Cronbach α of 0.955. EFA yielded a KMO value of 0.910, indicating robust sampling adequacy.

The PEU refers to the extent to which individuals perceive e-PHR services as easy and effortless to use, reflecting perceptions of ease in accessing and accepting the technology [46]. The measurement instrument was developed through the modification and refinement of items derived from the original constructs proposed by Venkatesh and Davis [45]. Responses were recorded using a 5-point Likert scale ranging from 1 (strongly disagree) to 5 (strongly agree). Higher scores indicated stronger perceptions of ease and convenience in using e-PHR services for health management. Previous research reported an internal consistency reliability (Cronbach α) of 0.902, and the reliability confirmed in this study was Cronbach α of 0.914, indicating excellent reliability. EFA yielded a KMO value of 0.836, suggesting strong sampling adequacy.

UI refers to an individual’s intention or commitment to accept and continuously use e-PHR services [47]. In this study, UI specifically addresses planned or intended future adoption and continued usage of e-PHR services. The measurement instrument was developed by adapting and refining items from the original constructs developed by Venkatesh et al [48]. Responses were measured on a 5-point Likert scale ranging from 1 (strongly disagree) to 5 (strongly agree). Higher scores indicated a greater intention to use e-PHR services. Previous research reported an internal consistency reliability (Cronbach α) of 0.936. The reliability confirmed in this study was also Cronbach α of 0.936, indicating excellent reliability. EFA yielded a KMO value of 0.874, suggesting strong sampling adequacy.

### Statistical Analysis

Descriptive statistics were used to summarize the participants’ general characteristics, while differences based on these characteristics were analyzed using 2-tailed independent *t* tests and ANOVA. The validity and reliability of the survey instrument were assessed through EFA and Cronbach alpha tests. Correlation analysis was conducted to investigate the relationships among the variables. Subsequently, the proposed research model was evaluated using SEM, and the model fit was assessed by examining indices sensitive to sample size and model parsimony, specifically the incremental fit index (IFI), Tucker–Lewis Index (TLI), comparative fit index (CFI), and root mean square error of approximation (RMSEA), and standardized root mean square residual. Mediation effects were tested using bootstrapping with 2000 resamples and bias-corrected 95% CIs, which allowed for the estimation of indirect, direct, and total effects associations among key constructs. Moderation effects were tested via multigroup SEM by comparing path coefficients between the mild and severe disability groups (severity defined by self-reported category). Statistical significance of between-group differences was evaluated using critical ratios for parameter differences. Prior to comparisons, configural and metric measurement invariance were examined to ensure meaningful cross-group tests.

Questionnaires that were clearly invalid, either blank pages or patterned straight-lining, were excluded prior to analysis. For item-level missing responses, descriptive statistics, correlations, and EFA used pairwise deletion; scale scores were computed when ≥50% of items in a scale were present, otherwise treated as missing. For SEM in AMOS, listwise deletion was applied to ensure stable ML estimation, consistent with common AMOS practice. Univariate outliers were screened using standardized *z* scores (|*z*|>3.29) and boxplots, and multivariate outliers using Mahalanobis distance based on all observed indicators (threshold: chi-square with *P*<.001 for the relevant df). Potential influence was examined in auxiliary ordinary least squares regressions for the main structural paths (leverage and Cook’s distance; rule-of-thumb>4/n). To assess common method bias, we estimated (1) a common latent factor (CLF; variance fixed to 1; equal method loadings) and (2) a marker-based unmeasured latent method construct (ULMC) model. Neither approach yielded material improvement in fit (CLF vs baseline: ΔCFI=0.002, ΔRMSEA=0.001; ULMC vs baseline: ΔCFI=0.003, ΔRMSEA=0.000), and standardized loadings and paths changed by less than 0.20. Thus, CMB is unlikely to bias the substantive conclusions.

Data processing and statistical analyses were performed using SPSS 23.0 (IBM Corp) and AMOS 23.0 (IBM Corp) software.

### Ethical Considerations

This study received ethics approval from the Korea University Institutional Review Board (KUIRB-2023-0286-01). Participants received information regarding the purpose, potential benefits, and risks of the study and were assured that all data would remain confidential. Each individual had the option to decline participation or withdraw from the study at any time. All participants signed an informed consent form. To protect anonymity and privacy, the data were encoded. To encourage participation, each respondent received a bathroom towel valued at US $5.

## Results

### General Characteristics

This study conducted a survey involving a total of 800 people with disabilities in Korea. The general characteristics of participants are summarized in Table 1. Regarding city size, 307 (38.4%) respondents resided in large metropolitan cities, while 493 (61.6%) were from small-to-medium-sized towns. The gender distribution indicated that males comprised a higher proportion (n=470, 58.8%) compared to females (n=330, 41.2%). The largest age group was 40‐49 years old (n=188, 23.5%), followed by the age groups of 50‐59 years and 60‐69 years, each at 20.3% (n=162). Regarding employment status, unemployed respondents (n=495, 61.9%) outnumbered those with employment (n=305, 38.1%). The educational background of the respondents showed that most had completed high school (n=383, 47.9%), followed by those with college degrees or higher (n=222, 27.8%). The duration since disability onset was highest for the group of 5 to less than 10 years (n=346, 43.3%), followed by less than 5 years (n=161, 20.1%). Disability severity showed a higher proportion of mild cases (n=432, 54%) compared to severe cases (n=368, 46%). Regarding marital status, unmarried respondents, including those who were widowed and divorced, accounted for a higher proportion (n=445, 55.6%) compared to married respondents (n=355, 44.4%). Institutions used by participants were hospitals (n=377, 30.3%), disability welfare centers (n=319, 25.7%), and local public health centers (n=278, 22.4%).

**Table 1.** General characteristics of the study (N=800).

Characteristics	Participants, n (%)
Region	
Metropolitan	307 (38.4)
Medium or small city	493 (61.6)
Sex	
Male	470 (58.8)
Female	330 (41.2)
Age group (years)	
20-29	96 (12)
30-39	120 (15)
40-49	188 (23.5)
50-59	162 (20.3)
60-69	162 (20.3)
70 or more	72 (9)
Employment status	
Employed	305 (38.1)
Unemployed	495 (61.9)
Educational level	
Elementary school or Less	77 (9.6)
Middle school	118 (14.8)
High school	383 (47.9)
College graduate or higher	222 (27.8)
Duration of illness (years)	
<5	161 (20.1)
5-10	346 (43.3)
10-15	126 (15.8)
15-20	43 (5.4)
20-25	51 (6.4)
25-30	7 (0.9)
30 or more	66 (8.3)
Disability grade	
Severe	368 (46)
Mild	432 (54)
Marital status	
Married	355 (44.4)
Single (including widowed, divorced, etc)	445 (55.6)
Service institutions used^a^	
Hospitals	377 (30.3)
Public health centers	278 (22.4)
Welfare centers for the disabled	319 (25.7)
Fitness facilities	216 (17.4)
Others	53 (4.3)

a multiple responses allowed.

### Correlation Matrix and Measurement Model

A correlation analysis of the measurement model was conducted to verify the causal relationships among key variables, and the presence of multicollinearity was evaluated by examining variance inflation factors (VIFs) and tolerance values. As shown in Table 2, the correlation matrix between the dependent variable, UI, and other study variables revealed statistically significant positive correlations with PU (*r*=0.780; *P*=.004), PEU (*r*=0.649; *P*=.005), HC (*r*=0.538; *P*=.005), IS (*r*=0.420; *P*=.002), EF (*r*=0.651; *P*=.005), CC (*r*=0.591; *P*=.003), HIC (*r*=0.616; *P*=.008), and eHL (*r*=0.323; *P*=.007).

**Table 2.** Correlation analysis among key variables.

Constructs	UI^a^	PU^b^	PEU^c^	HC^d^	IS^e^	EF^f^	CC^g^	HIC^h^	eHL^i^
UI									
*r*	1	0.780	0.649	0.538	0.420	0.651	0.591	0.616	0.323
*P* value	—	.004	.005	.005	.002	.005	.003	.008	.007
PU									
*r*	0.780	1	0.708	0.499	0.508	0.720	0.586	0.635	0.305
*P* value	.004	—	.002	.008	.003	.008	.006	.005	.006
PEU									
*r*	0.649	0.708	1	0.528	0.527	0.635	0.610	0.572	0.382
*P* value	.005	.002	—	.005	.003	.005	.005	.003	.008
HC									
*r*	0.538	0.499	0.528	1	0.448	0.542	0.481	0.418	0.307
*P* value	.005	.008	.005	—	.005	.007	.008	.008	.006
IS									
*r*	0.420	0.508	0.527	0.448	1	0.551	0.582	0.547	0.328
*P* value	.002	.003	.003	.005	—	.008	.008	.007	.007
EF									
*r*	0.651	0.720	0.635	0.542	0.551	1	0.687	0.612	0.416
*P* value	.005	.008	.005	.007	.008	—	.009	.005	.003
CC									
*r*	0.591	0.586	0.610	0.481	0.582	0.687	1	0.686	0.378
*P* value	.003	.006	.005	.008	.008	.009	—	.007	.004
HIC									
*r*	0.616	0.635	0.572	0.418	0.547	0.612	0.686	1	0.385
*P* value	.008	.005	.003	.008	.007	.005	.007	—	.004
eHL									
*r*	0.323	0.305	0.382	0.307	0.328	0.416	0.378	0.385	1
*P* value	.007	.006	.008	.006	.007	.003	.004	.004	—

a UI: usage intention.

b PU: perceived usefulness.

c PEU: perceived ease of use.

d HC: health consciousness.

e IS: information security.

f EF: effectiveness.

g CC: content characteristics.

h HIC: health information consent.

i eHL: eHealth literacy.

Correlation values among variables ranged from 0.344 to 0.772, all below the threshold of 0.90, while VIF values ranged from 1.296 to 2.921, all below the critical threshold of 10. Additionally, no tolerance values fell below 0.1, confirming the absence of multicollinearity. Criteria indicating the absence of multicollinearity include correlation coefficients among variables below 0.90, VIF values below 10, and tolerance levels above 0.1. The results of the structural model fit assessment are as follows: the chi-square value was calculated to be *χ*²_672_=2998.6, and model fit indices were IFI=0.929, TLI=0.922, CFI=0.929, and RMSEA=0.06. These values collectively indicate that the measurement model satisfactorily meets standard acceptance criteria, demonstrating good model fit.

Model refinement followed a prespecified, theory-first parsimony strategy. In AMOS, modification indices (MIs) were screened with MI≥10 and EPC≥0.10. Suggested residual covariances were freed only with a defensible common cause, otherwise left fixed. Indicators were considered for removal if λ<0.50 (implying <25% shared variance with the factor), |SR|>4, MI indicated cross-loading, or any Heywood case. Indicators producing a Heywood case, such as negative error variance or standardized loading greater than 1.0, were treated as inadmissible solutions and addressed through respecification; item parceling was not used. Alternative models were also estimated (the trimmed model dropping *P*>.10 paths, single-factor blocks, and higher-order variants). Model choice prioritized parsimony and information criteria alongside fit, with Bollen–Stine bootstrap (2000 resamples) to guard against overfitting. The final model retained a theory-congruent structure; MI-driven residual correlations without rationale were not adopted.

### Hypothesis Testing

The results of hypothesis testing are presented in Table 3. First, standardized path coefficients to PEU from HC (β=0.233; *P*<.001), CC (β=0.163; *P*<.001), HIC (β=0.167; *P*<.001), IS (β=0.089; *P*=.005), and EF (β=0.276; *P*<.001) were statistically significant. Conversely, the path from eHL (β=0.025; *P*=0.406) to PEU was not statistically significant, leading to the rejection of this hypothesis. Second, standardized path coefficients to PU from CC (β=–0.121; *P*<.001), HIC (β=0.243; *P*<.001), eHL (β=–0.068; *P*=.003), and EF (β=0.368; *P*<.001) were statistically significant. In contrast, paths from HC (β=0.049; *P*=0.135) and IS (β=–0.009; *P*=.77) to PU were not statistically significant and thus rejected. Third, regarding hypotheses involving PEU, PU, and UI, the standardized path coefficient from PEU to PU (β=0.452; *P*<.001) was statistically significant. Additionally, the standardized path coefficients from PU (β=0.662; *P*<.001) and PEU (β=0.203; *P*<.001) to UI were statistically significant, thus supporting all related hypotheses.

**Table 3.** Results of hypothesis testing among key variables.

Path	Estimate		CR (95% CI)^a^	Comments
	Unstandardized beta coefficient (β)	Standardized beta coefficient, β (SE)		
HC^b^→PEU^c^	0.318	0.233 (0.052)	6.107 (0.216 to 420)^d^	Supported
CC^e^→PEU	0.155	0.163 (0.043)	3.564 (0.071 to 0.239)^d^	Supported
HIC^f^→PEU	0.159	0.167 (0.039)	4.058 (0.083 to 0.235)^d^	Supported
eHL^g^→PEU	0.034	0.025 (0.04)	0.832 (–0.046 to 0.114)	Not supported
IS^h^→PEU	0.08	0.089 (0.032)	2.506 (0.018 to 0.142)^i^	Supported
EF^j^→PEU	0.269	0.276 (0.042)	6.436 (0.187 to 0.351)^d^	Supported
HC→PU^k^	0.08	0.049 (0.053)	1.494 (–0.024 to 0.184)	Not supported
CC→PU	–0.136	–0.121 (0.044)	–3.078 (–0.222 to –0.050)^i^	Supported
HIC→PU	0.276	0.243 (0.04)	6.821 (0.198 to 0.354)^d^	Supported
eHL→PU	–0.107	–0.068 (0.041)	–2.59 (–0.187 to –0.027)^i^	Supported
IS→PU	–0.009	–0.009 (0.032)	–0.29 (–0.072 to 0.054)	Not supported
EF→PU	0.424	0.368 (0.044)	9.715 (0.338 to 0.510)^d^	Supported
PEU→PU	0.534	0.452 (0.048)	11.204 (0.440 to 0.628)^d^	Supported
PU→UI^l^	0.62	0.662 (0.041)	15.201 (0.540 to 0.700)^d^	Supported
PEU→UI	0.225	0.203 (0.045)	5.019 (0.137 to 0.313)^d^	Supported

a CR: critical ratio.

b HC: health consciousness.

c PEU: perceived ease of use.

d *P*<.001

e CC: content characteristics.

f HIC: health information consent.

g eHL: eHealth literacy.

h IS: information security.

i *P*<.01

j EF: effectiveness.

k PU: perceived usefulness.

l UI: usage intention.

**Figure 2.**
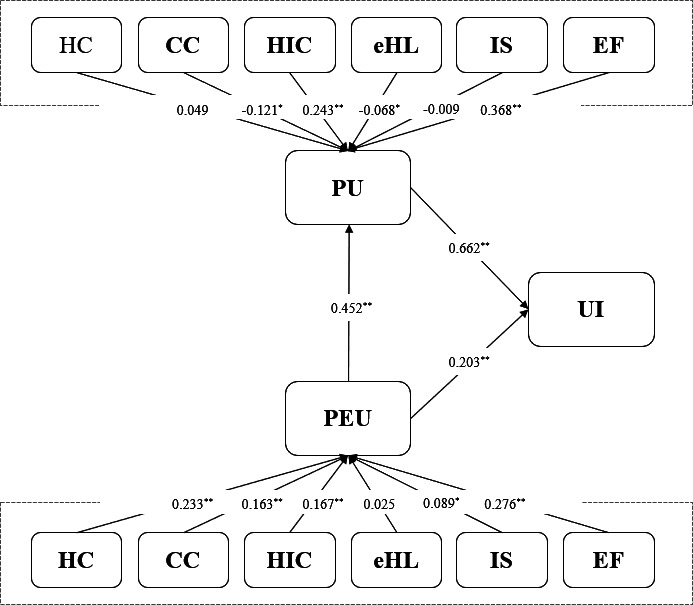
Structural equation model of factors influencing digital health technology adoption among people with disabilities. CC: content characteristics; EF: effectiveness; eHL: eHealth literacy; HC: health consciousness; HIC: health information consent; IS: information security; PEU: perceived ease of use; PU: perceived usefulness, UI: usage intention. *P<.01, ** *P*<.001

The structural model was validated, and causal relationships among latent variables were examined using SEM (Figure 2). HC had a significant positive effect on PEU (β=0.233; *P*<.001), which suggests that individuals with greater health concerns tend to find ePHRs more user-friendly. Additionally, CC has a notable impact on associations with PEU (β=0.163; *P*<.001), indicating that well-structured and easily understandable information enhances the user experience. Another important factor is the willingness to share personal health information (β=0.16; *P*<.001), which suggests that individuals who are open to sharing health data are more likely to find the system easier to accept. Although the standardized coefficient for IS awareness is relatively small (β=0.089; *P*=.005), it remains statistically significant. Among all predictors, the level of service support indicated the strongest associations with on PEU (β=0.276; *P*<.001). Users tend to find the system easier to navigate when comprehensive support and guidance are available. In contrast, eHL does not show a statistically significant relationship with PEU (β=0.025; *P*=0.406), leading to the rejection of the corresponding hypothesis.

When examining the factors that influence PU, CC was found to have a statistically significant negative impact (β=−0.121; *P*<.001), which suggests that overly abundant or complex content may lead users to view the system as less useful. In contrast, agreement with health information usage (β=0.243; *P*<.001) and the level of service support (β=0.368; *P*<.001) demonstrated significant positive associations, which indicates that users who perceive greater assistance from the ePHR system are more likely to consider it useful. Interestingly, eHL also had a statistically significant negative association with PU (β=−0.068; *P*=.003). This implies that users who are more familiar with digital health information may adopt a more critical perspective toward the system. On the other hand, HC (β=0.049; *P*=0.135) and IS awareness (β=−0.009; *P*=0.772) did not significantly influence PU, leading to the rejection of these 2 hypotheses.

Regarding the hypotheses addressing PEU, PU, and UI, the analysis revealed a statistically significant standardized path coefficient from PEU to PU (β=0.452; *P*<.001). Furthermore, the standardized path coefficients from PU (β=0.662; *P*<.001) and PEU (β=0.203; *P*<.001) to UI were also statistically significant. These findings suggest that particularly for special populations, such as individuals with disabilities, environmental factors like usability, reliability, and social support are more crucial for actual technology adoption than informational literacy. Therefore, future models of technology acceptance should integrate these multidimensional environmental factors into a comprehensive approach.

### Mediation Effect Analysis

Bootstrapping (2000 resamples; bias-corrected 95% CI) revealed the following mediation results (Table 4). First, HIC had significant positive direct effects on associations with PEU (β=0.167; *P*<.001) and PU (β=0.243; *P*<.001), and exerted a significant indirect effect on UI (total effect β=0.245; *P*<.001; Multimedia Appendix 2). Second, CC showed a significant positive association with PEU (β=0.163; *P*=.04) but a negative direct association with PU (β=−0.121, *p* = 0.345). When indirect effects were considered, the total effect on UI was negligible (β=0.002; *P*=.03). Third, IS exhibited a small positive association with PEU (β=0.089; *P*=.02) and, through PU, a limited indirect effect on UI (total effect β=0.039; *P*=.02). Fourth, EF had the strongest associations across all paths, with direct effects on PEU (β=0.276; *P*<.001) and PU (β=0.368, *P*<.001), yielding the largest total effect on intention to use when indirect effects were included (β=0.382; *P*<.001). Fifth, HC positively predicted PEU (β=0.233; *P*<.001) and contributed indirectly to intention to use via PU (total effect (β=0.150; *P*<.001). Finally, eHL did not show significant associations with PEU or PU and, if anything, trended negatively for PU (β=−0.068) and UI (β=−0.032).

**Table 4.** Bootstrapped indirect effects.

Indirect path	β indirect (95% CI)	*P* value
PEU^b^ → PU^c^ → UI^d^	0.299 (0.210-0.404)	<.001
HIC^e^ → PU → UI	0.245 (0.178-0.326)	<.001
EF^f^ → PU → UI	0.382 (0.301-0.470)	<.001
HC^g^ → PU → UI	0.15 (0.098-0.215)	<.001
IS^h^ → PU → UI	0.039 (0.014-0.075)	.01
CC^i^ → PU → UI	0.002 (0.001-0.007)	.04
eHL^j^ → PU → UI	–0.032 (–0.089-0.014)	.18

a BC: bootstrapping corrected.

b PEU: perceived ease of use.

c PU: perceived usefulness.

d UI: usage intention.

e HIC: health information consent.

f EF: effectiveness.

g HC: health consciousness.

h IS: information security.

i CC: content characteristics.

j eHL: eHealth literacy.

### Moderation Effect Analysis

Multigroup SEM (mild n=432; severe n=368) showed that HIC and EF were positively associated with PEU and PU in both groups (all *P*<.001; Multimedia Appendix 3). CC predicted PEU only in the mild group (β=0.201; *P*<.001), while IS predicted PEU only in the severe group (β=0.119; *P*=0.003). For PU, HIC and PEU were positively associated with both groups, whereas eHL was negatively associated with the mild group (β= −0.074; *P*=.006) and CC was also negatively associated with the mild group (β= −0.215; *P*<.001). UI was driven primarily by PU in both groups (mild β=0.727; severe β=0.511; both *P*<.001), with an additional contribution from PEU (severe β=0.272; mild β=0.171; *P*<.001).

## Discussion

### Determining Effect of PU and Ease of Use

In this study, PU emerged as the strongest predictor of the intention to use digital health technologies. The findings suggest that the adoption of digital health technologies among people with disabilities is more likely when there is a strong belief in the genuine benefits of these technologies for health management activities [24]. Furthermore, the study by Holden and Karsh [49] also emphasized the applicability and robustness of the TAM within health care contexts. For instance, Harrison et al [50] reported that 69.8% of patients with chronic kidney disease expressed a willingness to use digital technologies because perceived benefits include increased involvement in treatment and easier access to laboratory results. Similarly, Khor et al [51] noted that PU significantly affects user attitudes toward e-PHR, positively impacting subsequent intentions to use these services. These findings suggest that the adoption of digital health technologies is driven not only by functional capabilities but also by perceptions of tangible, practical benefits, and that establishing trust in the effectiveness and practical value of digital health technologies is essential for this population.

PEU also significantly influenced users’ intention to adopt digital health technologies. This finding aligns with Pai and Huang [52], who emphasized that minimizing technological complexity is crucial in forming UIs, particularly during the initial stages of implementing a hospital information system. Similarly, Tavares and Oliveira [53] identified PEU as a direct determinant of adoption intentions in their study of electronic medical record portals. Additionally, a systematic literature review by Rahimi et al [54] consistently confirmed that PEU significantly impacts the intention to use health informatics systems, acting as a key factor in reducing psychological resistance associated with technological complexity, especially during the early stages of system adoption. Akritidi et al [55] further highlighted that intentions to use digital health care services are substantially influenced by PU, PEU, user satisfaction, privacy and security considerations, user age, and familiarity with electronic services.

Additionally, PEU was found to have a positive influence on PU. This finding suggests that as technology is perceived as intuitive and easy to use, the expectation that it will substantially benefit health management activities increases. These results align with empirical evidence provided by Yarbrough and Smith [56], who demonstrated that medical professionals are more likely to perceive systems as applicable when those systems are uncomplicated to use. Similarly, Aggelidis and Chatzoglou [57] empirically confirmed the significant influence of ease of use on PU in studies concerning hospital information systems and the acceptance of health IT among physicians. Chau and Hu [58] further emphasized that PEU serves as a crucial mediating factor affecting PU and behavioral intentions in telemedicine and nursing information systems. A meta-analysis also highlighted this relationship as one of the most consistently validated paths across various TAM-based studies [59]. Consequently, ease of use emerges as an especially critical factor for user groups, such as people with disabilities, who often face substantial barriers to information access. Thus, when designing digital health technologies, strategies that prioritize user-friendly interfaces, such as clear visual displays, simple navigation, and step-by-step instructions, should be emphasized. For technologies targeting users with disabilities specifically, incorporating features, such as assistive device compatibility, voice-guided instructions, and adherence to accessibility standards is essential [60-62].

### Factors Influencing Ease of Use

Factors significantly influencing the PEU included HC, CC, HIC, IS, and EF. Notably, the EF exerted the strongest influence, empirically demonstrating the crucial role of social support and facilitating conditions in the acceptance process of digital health technologies. According to Venkatesh et al [63], social influence and facilitating conditions were key determinants in technology acceptance, with expectations and support from others significantly increasing an individual’s intention to adopt technology. Similarly, Heart and Kalderon [64] emphasized that ongoing support from family members, professionals, or caregivers substantially facilitates technology acceptance among vulnerable populations, such as people with disabilities and older adults.

Furthermore, this study illustrated that the positive impact of HC on health management significantly impacts actual technology usage behavior, which aligns with findings by Or and Karsh [65], who highlighted personal health interest and motivation as critical antecedents of eHealth technology acceptance, demonstrating that individuals with greater health motivation are more inclined to adopt such technologies readily. Additionally, research by Cocosila and Archer [66] confirmed that individuals who perceive health improvement as a primary goal tend to accept and positively evaluate mobile health technologies more easily.

CC also significantly influenced PEU, indicating that the composition of content, presentation style, and degree of information structuring impact user intuitiveness and satisfaction with technology use. This finding aligns with Zhang and von Dran [67], who demonstrated that users commonly assess the quality of web-based technologies based on the visual arrangement of information, ease of navigation, and the interconnected structure of content. Additionally, Liu and Shrum [68] reported that how the presentation of this information affects cognitive load and user engagement, emphasizing that visual and interactive content can enhance ease of understanding compared to text-centric approaches. Crutzen et al [69] similarly emphasized that visual design and information delivery methods in web-based health information systems substantially affect not only user usability but also learning outcomes.

It is also noteworthy that consent to share health information significantly influences the PEU. When users voluntarily consent to provide health information, it fosters trust in technology, which can potentially impact the overall PEU. This result aligns with the findings of Bansal et al [70], who emphasized that individuals were comfortable using digital platforms to share sensitive health data only when the users trusted the platforms’ privacy protection mechanisms. Similarly, Li [71] reported that users who depend on a system’s security features experience reduced psychological resistance, which in turn leads to more positive evaluations of the technology’s ease of use.

Finally, IS was also found to have a significant influence on PEU, which suggests that when personal data are perceived as securely protected, psychological anxiety about technology usage decreases, allowing for more intuitive system usage. These findings align with those of Bansal et al [70], who emphasized that trust in privacy protection is a fundamental prerequisite for accepting technologies that involve sharing sensitive health information. Similarly, Angst and Agarwal [72] showed that in the context of electronic medical record system adoption, building trust in data protection reduces resistance to technology and enhances intuitive and secure system usage. Klaver et al [73] further reported that trust in security directly affects users’ psychological comfort and perceived ease of using mobile health technologies, highlighting that it is especially critical for older adults and vulnerable populations.

### Factors Influencing PU

Factors identified as influencing PU included the EF, HIC, CC, and eHL. Most importantly, the EF had the strongest influence, suggesting that for users with physical disabilities, technology’s effectiveness is evaluated not merely based on its functional attributes but also significantly through social interactions and support experienced within the given environment. This result aligns with findings from Holden and Karsh [49], who reported that users’ perceptions of health care technology acceptance are significantly influenced by external factors, such as facilitators or environmental conditions, rather than solely by individual judgments. Similarly, Chen and Chan [74] demonstrated that support from family members or caregivers plays a crucial role in older adults’ recognition of the practical utility of technology.

Additionally, the finding that consent to share health information positively influences PU suggests that voluntarily agreeing to provide personal health data fosters trust in technology and enhances users’ sense of autonomy. These psychological factors, in turn, contribute significantly to the overall evaluation of the technology’s usefulness. This interpretation aligns with the findings of Bansal et al [70], who reported that users’ perceptions of technology usefulness improve when trust in privacy protection and a sense of personal information control are established, particularly when dealing with sensitive health data. Likewise, Li [71] noted that the voluntary provision of information enhances psychological comfort, positively influencing overall user evaluations of technology. Angst and Agarwal [72] also empirically demonstrated that maintaining autonomy in information disclosure during the adoption of an electronic health record system critically influences user trust in the technology and subsequent PU.

The negative associations between content richness and eHL with PU may be consistent with cognitive-load and information-overload accounts. When information volume or complexity exceeds users’ processing capacity, it may become harder to extract actionable value, potentially lowering PU [75]. For higher-literacy users, expectation–disconfirmation processes may also contribute [76]. When these expectations are not met, evaluations of usefulness decline, even if users understand the content. This trend aligns with findings indicating that higher eHL can lead to more critical assessment and lower perceived utility, particularly when quality indicators are weak [77-79]. Conversely, several studies suggest a positive relationship between literacy and PU when the quality of content is demonstrably strong [80,81]. To address these issues, designers should avoid a one-size-fits-all approach to content richness and instead implement layered content and highlight quality indicators, such as sources, levels of evidence, and personalization logic, especially for users with high literacy. These strategies can help reconcile the negative associations observed in research with theoretical frameworks and suggest actionable steps to enhance PU across different literacy levels.

On the other hand, CC and eHL were each found to have a negative influence on PU. This suggests that users with higher information interpretation skills may react more sensitively to perceived qualitative limitations or technological shortcomings in content. Similar findings were reported by Chung and Nahm [77], who indicated that users with higher eHL tend to critically assess the reliability, accuracy, and personalization of content, which can negatively affect the perceptions of its usefulness. Also, studies by Diviani et al [82] and Neter and Brainin [79] emphasized that higher literacy increases user expectations and criteria for information quality, potentially resulting in diminished perceptions of the technology if these expectations are not met. Conversely, research by Norman and Skinner [80] and Mackert et al [81] has demonstrated that increased literacy enhances information-seeking abilities, thereby improving the PU and acceptance of digital technologies.

### Mediation and Moderation Effects of Technology Acceptance in People With Disabilities

Applying a TAM-based model, the study examined the determinants of digital health service acceptance among individuals with disabilities and analyzed the mediation effects as well as the moderation by disability severity. First, mediation analyses identified PEU as a central mediator within the model. The HIC significantly increased both PEU and PU and, via PU, exerted a positive indirect effect on the intention to use. This suggests that transparency and trust in the use of personal information are foundational factors for health care technology acceptance [83,84] and should be emphasized in services designed for individuals with disabilities. In addition, the EF showed the strongest explanatory power across all paths, confirming that social and professional support are decisive drivers of technology adoption—a finding consistent with prior reports underscoring the importance of support systems for vulnerable populations [85,86].

Second, not all predictors exerted uniformly positive effects. CC had a positive effect on PEU but showed a nonsignificant negative association with PU, resulting in a minimal total effect on intention to use. This pattern suggests that informational richness does not automatically translate into usefulness, indicating that groups with extensive digital experience may evaluate system quality more critically [87]. IS positively influenced the PEU yet had a limited direct effect on the intention to use, implying that security functions as a necessary but not sufficient condition for adoption [88]. eHL did not exhibit a positive association with either PEU or PU, instead trending negatively. A plausible interpretation is that higher-literacy users were more sensitive to simplicity or the lack of personalization in the service. This is consistent with Norman and Skinner’s [80] eHealth Literacy framework, which suggests that elevated literacy prioritizes information quality and sophistication, potentially heightening dissatisfaction with comparatively simple systems.

Third, moderation analyses by disability severity revealed between-group differences among several key paths. HC and EF were consistently significant in both the mild and severe groups, whereas CC was significant only in the mild group, and IS was significant only in the severe group. In addition, eHL had a negative association with PU in the mild group. The above patterns suggest that individuals with milder disabilities, who generally possess higher digital capability, tend to evaluate systems more critically, while individuals with more severe disabilities place greater emphasis on accessibility and security [89]. In both groups, PEU emerged as the strongest determinant of intention to use, consistent with prior TAM evidence [48]. The association with PU was larger in the severe group, indicating that the practical benefits of technology carry greater weight in acceptance decisions among those with more severe disabilities [90].

The finding that eHL had a negative association with PEU in the mild group can be interpreted in several ways. First, differences in evaluative standards may play a role. Individuals in the mild group typically have greater digital access and more extensive online information experience, which fosters more critical appraisal of system quality, accuracy, and convenience. In contrast, individuals in the severe group often face constrained digital options; even with higher literacy, accessibility and availability of external support may be prioritized over ease of use, attenuating any literacy–ease relationship [91]. Second, expectation–disconfirmation theory offers a complementary explanation. When a system is relatively simple or lacks personalization, unmet expectations may lead to depressed ease-of-use judgments. Conversely, for the severe group, basic accessibility itself is paramount, and unmet expectations may have been less salient in this domain [92].

### Limited Role of eHL

In this study, eHL did not significantly influence PEU, but it was negatively associated with PU. Similar results were reported by Chung and Nahm [77], who indicated that among older adults, even high levels of eHL could not compensate for inadequate system accessibility and ease of use, consequently limiting technology acceptance. Moreover, van der Vaart et al [93] emphasized that adequate technological infrastructure and ongoing support conditions must accompany information interpretation abilities to translate literacy into actual technology use. Czaja et al [94] further demonstrated that environmental factors, tool accessibility, and social support are more critical determinants of technology acceptance than cognitive capabilities alone.

The aforementioned findings suggest that, particularly among groups such as people with disabilities, factors such as physical accessibility, ease of device use, and the presence of social support are more critical determinants of actual technology acceptance than literacy alone. Having said that, there is a vital need for integrated model designs that comprehensively incorporate environmental, social, and policy-related factors surrounding the adoption of technology [95].

### Limitations

While the study yielded promising results, several important limitations should be acknowledged. First, the study was conducted among individuals accessing specific institutions, such as rehabilitation hospitals, welfare centers for people with disabilities, and public health centers. This focus limits the generalizability of the findings to all people with disabilities, as the sample does not include those living in diverse environments. Future research should aim to include broader samples that consider various factors, such as residential settings, levels of community participation, and access to information resources. Second, the study used a cross-sectional design, which limits the ability to draw clear causal relationships between variables. Therefore, conducting longitudinal studies that track the same group of participants over time is recommended to clarify temporal causality among variables [96]. Third, data collection relied solely on self-report questionnaires. It could introduce social desirability bias, as participants may respond in ways they believe to be socially acceptable rather than reflecting actual behaviors. For the study, to address this issue, on-site paper surveys were conducted in private rooms, allowing unlimited time for responses. Emphasis was placed on anonymity and aggregate reporting, with neutral instructions provided. When assistance was needed, staff read the survey items verbatim and recorded responses without offering evaluative feedback. Any remaining bias is expected to elevate response levels rather than alter observed associations, indicating that the negative or null relationships noted are unlikely to be artifacts. Future studies should incorporate a brief social desirability scale or use indirect questions, enhance the use of private self-administered methods, and adjust models to account for the mode of administration. Fourth, this study analyzed people with disabilities as a pooled group, which improves statistical stability but limits granularity with respect to disability type and severity. Heterogeneity across these dimensions may shape both baseline levels and structural relations. Small subgroup counts and the absence of established measurement invariance precluded reliable multigroup estimation in the present data. Conducting such analyses without adequate power risks overfitting and spurious differences. Future research should use stratified sampling to ensure sufficient cases and consider hierarchical models to estimate between-group variance components [97]. Practically, intervention design should anticipate heterogeneity by offering layered content and adaptable interfaces and by segmenting onboarding/support according to severity. These steps will enable more actionable, subgroup-specific recommendations while maintaining psychometric rigor.

### Practical Implications for Policy, System Design, and Clinical Implementation

Beyond the theoretical validation of TAM constructs, the present findings offer concrete implications for disability-specific digital health strategies. At the policy level, governments should establish national accessibility standards for e-PHR platforms, expand digital literacy programs for people with disabilities, and allocate funding to community-based support systems to reduce disparities. From a system design perspective, developers should incorporate accessibility features such as screen readers, voice navigation, and simplified interfaces tailored to cognitive or sensory impairments, ideally through participatory co-design with people with disabilities. Clinically, healthcare providers should integrate e-PHR services into rehabilitation and chronic care workflows, deliver clinician-guided onboarding sessions, and ensure interoperability with assistive devices to maximize usability and trust. To operationalize service support, it may help to establish assisted onboarding services within hospitals and welfare centers, integrate caregiver-managed accounts for those with cognitive or motor impairments, and simplify content presentation to accommodate users with low health or digital literacy. For individuals with severe disabilities, priority should be given to caregiver-managed accounts, voice-navigation features, and continuous assisted support. For those with mild disabilities, simplified user interfaces, standardized accessibility tools, and short-term digital literacy training may be sufficient.

### Conclusion

This study examined the factors influencing the intention to use digital health technologies among people with disabilities through the lens of the TAM and analyzed the structural relationships among relevant variables. The findings revealed that both PU and PEU were significantly associated with the intention to use these technologies. Several external factors, including EF, HIC, and CC, also influenced these mediating variables. Notably, the EF demonstrated the strongest associations with both PEU and PU, emphasizing the crucial role of social support in the technology acceptance process for people with disabilities. Future research should explore longitudinal studies and incorporate mixed methods approaches to further validate these findings and gain deeper insights into long-term acceptance and real-world usage behaviors among diverse people with disabilities. Overall, this study provides valuable insights into developing digital health services and informs policy-making efforts specifically tailored for people with disabilities.

## Supplementary material

10.2196/79595 Multimedia Appendix 1 Survey instruments.

10.2196/79595 Multimedia Appendix 2 Mediation effect analysis.

10.2196/79595 Multimedia Appendix 3 Moderation effect analysis.

## Abbreviations

AMOS: Analysis of Moment Structures
CC: content characteristics
CFI: comparative fit index
CLF: common latent factor
e-PHR: electronic personal health record
EF: effectiveness
EFA: exploratory factor analysis
eHL: eHealth Literacy
HC: health consciousness
HIC: health information consent
HIMSS: Healthcare Information and Management System Society
IFI: incremental fit index
IS: information security
KMO: Kaiser-Meyer-Olkin
MI: modification index
MMSE: Mini-Mental State Examination
PEU: perceived ease of use
PU: perceived usefulness
RMSEA: root mean square error of approximation
SEM: structural equation modeling
TAM: Technology Acceptance Model
TLI: Tucker–Lewis Index
UI: usage intention
ULMC: unmeasured latent method construct
VIF: variance inflation factor
