# Supplementary material for: Using a Technology Acceptance Model to Explore the Intention to Use Digital Health Technologies Among People With Disabilities: Cross-Sectional Survey Study
Source: J Med Internet Res. 2025 Nov 20;27:e79595. doi: 10.2196/79595 (PMC12634014; doi:10.2196/79595)
Supplement: Multimedia Appendix 1 [file jmir-v27-e79595-s001.docx]

**1. The following questions relate to the intention to use digital healthcare (Personal Health Record) services: Usage Intention**

☐ Please check the corresponding number.

| **Response Scale** | **① Not at all** | **② Not really** | **③ Neutral** | **④ Somewhat agree** | **⑤ Strongly agree** |
| --- | --- | --- | --- | --- | --- |

**Statements:**

1. I intend to use personal health record services in the future.
2. I would recommend personal health record services to others in the future.
3. I intend to continuously use personal health records to manage my health.
4. I intend to manage my health by visiting institutions that operate personal health records (e.g., hospitals, health centers, welfare centers, etc.).
5. I am willing to try using personal health record services through digital means such as smartphones, mobile apps, and wearable devices.

**2. The following questions relate to the Perceived Usefulness of digital healthcare (Personal Health Record) services.**

☐ Please check the appropriate number.

| **Response Scale** | **① Not at all** | **② Not really** | **③ Neutral** | **④ Somewhat agree** | **⑤ Strongly agree** |
| --- | --- | --- | --- | --- | --- |

**Statements:**

1. Personal health record services will be generally beneficial to my life.
2. Personal health record services will fulfill my health needs.
3. Personal health record services will help improve my health management.
4. Personal health record services will help prevent secondary disabilities.
5. Through personal health records, I will be able to consistently and independently manage my health in the local community.
6. Personal health record services will reduce waiting times at institutions such as hospitals, health centers, and welfare centers for persons with disabilities.
7. Through the exchange of information between hospitals, health centers, and disability welfare institutions that provide personal health record services, I will be able to receive a variety of services and manage my health in a more integrated way.

**3. The following questions relate to the Perceived Ease of Use of digital healthcare (Personal Health Record) services.**

☐ Please check the appropriate number.

| **Response Scale** | **① Not at all** | **② Not really** | **③ Neutral** | **④ Somewhat agree** | **⑤ Strongly agree** |
| --- | --- | --- | --- | --- | --- |

**Statements:**

1. Using personal health record services will make health management more convenient.
2. Using personal health record services will allow me to easily receive the health information I want.
3. The method of using personal health record services will not be difficult.
4. It will not be difficult to use personal health record services alone.
5. It will not be difficult to use the related institutions that provide personal health record services, such as hospitals, health centers, and welfare centers for the disabled.

**4. The following questions relate to Health Consciousness.**

☐ Please check the appropriate number.

| **Response Scale** | **① Not at all** | **② Not really** | **③ Neutral** | **④ Somewhat agree** | **⑤ Strongly agree** |
| --- | --- | --- | --- | --- | --- |

**Statements:**

1. I tend to exercise for my health.
2. I tend to regularly check my health.
3. I tend to seek out health-related information through books, TV, the Internet, etc.
4. I tend to take health supplements for my health.
5. I tend to be selective about what I eat for health purposes.
6. I regularly undergo health checkups.
7. I am currently engaged in activities like fitness, yoga, etc., for my health.
8. I think health is very important in my life.

**5. Do you think the information security of digital healthcare (Personal Health Record) services is safe for managing the health of persons with disabilities?**

☐ Please check the appropriate number.

| **Response Scale** | **① Not at all** | **② Not really** | **③ Neutral** | **④ Somewhat agree** | **⑤ Strongly agree** |
| --- | --- | --- | --- | --- | --- |

**Statements:**

1. I believe that personal information is generally safe when using health management services based on personal health records.
2. I believe the level of information security is generally safe when using health management services based on personal health records.
3. I believe that personal health record information will not be leaked when shared across multiple institutions.
4. I believe that personal health records provided by health management services will protect the confidentiality of personal information.

**6. To what extent do you think digital healthcare (Personal Health Record) services are helpful for the health management of persons with disabilities?: Effectiveness**

☐ Please check the appropriate number.

| **Response Scale** | **① Not helpful at all** | **② Not helpful** | **③ Neutral** | **④ Helpful** | **⑤ Very helpful** |
| --- | --- | --- | --- | --- | --- |

**Items:**

1. Quick response to emergency situations
2. Management of health risk factors (e.g., alcohol, smoking, eating habits)
3. Communication between persons with disabilities and caregivers
4. Information sharing and integrated management of personal health status
5. Tracking of consultation details
6. Time management
7. Reduction in medical expenses
8. Continuous exercise management
9. Continuous personal health management
10. Prevention of secondary disabilities
11. Management of rehabilitation and treatment schedules
12. Social reintegration after rehabilitation
13. Community-based health management
14. Monitoring of routine basic examinations
15. Personalized health information provision and education
16. Support for inter-institutional linkage functions

**7. Do you think the content characteristics of digital healthcare (Personal Health Record) services are helpful for the health management of persons with disabilities?**

☐ Please check the appropriate number.

| **Response Scale** | **① Not helpful at all** | **② Not helpful** | **③ Neutral** | **④ Helpful** | **⑤ Very helpful** |
| --- | --- | --- | --- | --- | --- |

**Statements:**

1. Health management services using personal health records will be helpful for managing my own health.
2. Health management services using personal health records will be provided in a customized format, considering the type and severity of disability.
3. Health management services using personal health records can be used by me anywhere.
4. Personal health record services can be used while traveling.
5. Health management services using personal health records can be used appropriately in any situation.
6. Health management services using personal health records can be used anytime I need them.

**8. Are you willing to consent to the use of your health information for the provision of digital healthcare (Personal Health Record) services?**

☐ Please check the appropriate number.

| **Response Scale** | **① Strongly Disagree** | **② Disagree** | **③ Neutral** | **④ Agree** | **⑤ Strongly Agree** |
| --- | --- | --- | --- | --- | --- |

**Statements:**

1. I agree to provide my personal health record information for public purposes (e.g., hospitals, public health centers, welfare centers for persons with disabilities).
2. I agree to provide my personal health record information for research aimed at improving health management for persons with disabilities.
3. I agree to provide my personal health record information to medical-related technology companies (e.g., pharmaceutical or device companies).
4. I agree to provide my personal health record information to my insurance company.
5. I agree to provide my personal health record information to institutions for system development or inter-institutional sharing.
6. I agree to provide my personal health record information to my family, caregivers, or activity assistants.
7. I agree to provide my personal health record information to a multidisciplinary team of experts for health management.
8. I agree to storing my personal health record information in the cloud (virtual storage).

**9. Please respond based on your usual thoughts and experiences regarding eHealth Literacy in using digital healthcare (Personal Health Record) services for the health management of persons with disabilities.**

☐ Please check the appropriate number.

| **Response Scale** | **① Not at all** | **② Not really** | **③ Neutral** | **④ Agree** | **⑤ Strongly agree** |
| --- | --- | --- | --- | --- | --- |

**Statements:**

1. I need help from people around me to write health-related documents (e.g., vaccination records, medical records, health check-up reports) using the internet or mobile devices.
2. I can find information on the Internet to solve health problems.
3. There are health-related terms I do not understand.
4. It is difficult to understand information shared or explained by others.
5. I can find the health information I need in a short amount of time.
6. I know where the health information I need is provided.
7. I can find information related to healthy lifestyle habits using the internet or mobile devices.
8. I can understand medical explanations (disease, symptoms, etc.) and instructions provided on the internet.
9. I can discuss my health status with others.
10. I can ask appropriate questions when talking about my health.
11. I can share opinions when participating in group discussions or forums related to health.
12. I can convey my health-related opinions to professionals.
13. I can talk to professionals other than doctors about health.
14. I can share the health information I collect through blogs or social media (SNS).
