# Supplementary material for: Using a Technology Acceptance Model to Explore the Intention to Use Digital Health Technologies Among People With Disabilities: Cross-Sectional Survey Study
Source: J Med Internet Res. 2025 Nov 20;27:e79595. doi: 10.2196/79595 (PMC12634014; doi:10.2196/79595)
Supplement: Multimedia Appendix 2 [file jmir-v27-e79595-s002.docx]

**Multimedia Appendix 2**. Mediation effect analysis. HC: health consciousness, CC: content characteristics, HIC: health information consent, eHL: eHealth literacy, IS: information security, EF: effectiveness, PU: perceived usefulness, PEU: perceived ease of use, UI: usage intention.

| **Path** | **Direct effect** | **Indirect effect** | **Total effect** |
| --- | --- | --- | --- |
| HIC → PEU | 0.167 | 0 | 0.167*** |
| HIC → PU | 0.243 | 0.075 | 0.319*** |
| HIC → UI | 0 | 0.245 | 0.245*** |
| CC → PEU | 0.163 | 0 | 0.163* |
| CC → PU | -0.121 | 0.074 | -0.047 |
| CC → UI | 0 | 0.002 | 0.002* |
| IS → PEU | 0.089 | 0 | 0.089* |
| IS → PU | -0.009 | 0.04 | 0.032* |
| IS → UI | 0 | 0.039 | 0.039* |
| EF → PEU | 0.276 | 0 | 0.276*** |
| EF → PU | 0.368 | 0.125 | 0.492*** |
| EF → UI | 0 | 0.382 | 0.382*** |
| HC → PEU | 0.233 | 0 | 0.233*** |
| HC → PU | 0.049 | 0.105 | 0.155*** |
| HC → UI | 0 | 0.15 | 0.15*** |
| eHL → PEU | 0.025 | 0 | 0.025 |
| eHL → PU | -0.068 | 0.012 | -0.056 |
| eHL → UI | 0 | -0.032 | -0.032 |

**p* < 0.05, ***p* < 0.01, ****p* < 0.001
