# Supplementary material for: Using a Technology Acceptance Model to Explore the Intention to Use Digital Health Technologies Among People With Disabilities: Cross-Sectional Survey Study
Source: J Med Internet Res. 2025 Nov 20;27:e79595. doi: 10.2196/79595 (PMC12634014; doi:10.2196/79595)
Supplement: Multimedia Appendix 3 [file jmir-v27-e79595-s003.docx]

**Multimedia Appendix 3**. Moderation effect analysis. B: unstandardized coefficient, t(Sig.): t-statistic (with its associated Significance level), HC: health consciousness, CC: content characteristics, HIC: health information consent, eHL: eHealth literacy, IS: information security, EF: effectiveness, PU: perceived usefulness, PEU: perceived ease of use, UI: usage intention.

| **Path** | **Severe** | | | **Mild** | | |
| --- | --- | --- | --- | --- | --- | --- |
|  | **B** | ***β*** | **t(Sig.)** | **B** | ***β*** | **t(Sig.)** |
| HC → PEU | 0.306 | 0.247 | 4.119*** | 0.331 | 0.227 | 4.475*** |
| CC → PEU | 0.106 | 0.111 | 1.547 | 0.191 | 0.201 | 3.334*** |
| HIC → PEU | 0.158 | 0.187 | 3.097** | 0.16 | 0.154 | 2.722** |
| eHL → PEU | 0.059 | 0.056 | 1.113 | 0.022 | 0.014 | 0.362 |
| IS → PEU | 0.099 | 0.119 | 2.241** | 0.058 | 0.061 | 1.223 |
| EF → PEU | 0.226 | 0.232 | 3.299*** | 0.293 | 0.294 | 5.285*** |
| HC → PU | 0.038 | 0.03 | 0.566 | 0.151 | 0.084 | 1.925* |
| CC → PU | 0.045 | 0.046 | 0.738 | -0.253 | -0.215 | -4.181*** |
| HIC → PU | 0.222 | 0.257 | 4.756*** | 0.314 | 0.245 | 5.064*** |
| eHL → PU | -0.026 | -0.024 | -0.542 | -0.143 | -0.074 | -2.231** |
| PEU → PU | 0.429 | 0.421 | 6.648*** | 0.585 | 0.474 | 9.735*** |
| IS → PU | -0.058 | -0.069 | -1.475 | 0.033 | 0.028 | 0.674 |
| EF → PU | 0.253 | 0.255 | 4.097*** | 0.462 | 0.376 | 7.724*** |
| PU → UI | 0.549 | 0.511 | 7.199*** | 0.645 | 0.727 | 13.148*** |
| PEU → UI | 0.297 | 0.272 | 3.946*** | 0.187 | 0.171 | 3.392*** |

**p* < 0.05, ***p* < 0.01, ****p* < 0.001
